# When and why are the elderly medical patients admitted and readmitted?

**DOI:** 10.1186/1757-7241-22-S2-A56

**Published:** 2015-07-16

**Authors:** Donna L Wolff, Christian B Mogensen

**Affiliations:** 1 Medical Center, Hospital of Southern Denmark, Aabenraa, Denmark; 2 Emergency Center, Hospital of Southern Denmark, Aabenraa, Denmark

## Background

Acute hospitalizations of elderly people are increasing, resource-demanding, and potentially harmful. There is an ongoing interest in alternatives to admissions and in reducing the length-of-stay. Before suggesting alternatives more knowledge is requested, especially concerning admission patterns and whether short stay is associated with increased risk of re-admission. The aim was to describe the acute medical (re)admission patterns in patients more than 65 years old.

## Methods

A retrospective cohort study included all 65+ years acute medical admissions between April 2012 - February 2014 in 3 hospitals of Southern Jutland. Data about patient and hospital characteristics was analyzed using logistic regression. Primary admission was defined as no previous admission within the last 30 days and readmission as less than 30 days since last admission.

## Results

Preliminary results showed that among 65+ years 15,714 acute medical admissions accounted for 37% of all acute adult hospital admissions. The median age was 78 years (q25-q75: 71-84 years) equally gender distributed. The admission rate was significantly higher in January and July (9%) and lower in April (7%) than the average months (p < 0.0001). Monday and Friday had the highest and Saturday and Sunday the lowest admission (16% vs 11%, p < 0.0001). 81% were admitted between 8 am. and 21 pm. Median length-of-stay was 2.7 days (q25-q75: 0.9 - 6.3 days). 44% were discharged within 48 hours and only 22% stayed for more than 1 week. 19% of the admissions were readmissions, 16% after a primary admission. There was a significant lower readmission rate after short versus long (<> 48 hours) primary admissions (13% vs. 18% p < 0.0001). Increased risk for readmission was also related to gender and month of admission, but not to the patient's municipal, triage, age, diagnosis, hospital, department shifts, weekday, or time of primary admission.

## Conclusions

Medical acute admissions among elderly account for more than 1/3 of all acute admissions among adults and occur mainly during daytime. 19% are readmissions, and the risk of readmission were not associated to short in-hospital stay, age, municipal, or discharge diagnosis but to month of admission and gender.

